# Framework for integrating electronic patient-reported data in routine cancer care: an Oncology Intake Questionnaire

**DOI:** 10.1093/jamiaopen/ooac064

**Published:** 2022-07-26

**Authors:** Nadine J McCleary, Ellana K Haakenstad, Jessica L F Cleveland, Michael Manni, Michael J Hassett, Deb Schrag

**Affiliations:** Medical Oncology, Dana-Farber Cancer Institute, Boston, Massachusetts, USA; Medical Oncology, Dana-Farber Cancer Institute, Boston, Massachusetts, USA; Information & Analytics, Dana-Farber Cancer Institute, Boston, Massachusetts, USA; Information & Analytics, Dana-Farber Cancer Institute, Boston, Massachusetts, USA; Medical Oncology, Dana-Farber Cancer Institute, Boston, Massachusetts, USA; Medical Oncology, Dana-Farber Cancer Institute, Boston, Massachusetts, USA

**Keywords:** patient-reported outcomes, patient-reported data, electronic patient-reported outcomes, intake questionnaire, questionnaire implementation

## Abstract

**Objective:**

As part of ongoing implementation of electronic patient-reported outcome tools at the Dana-Farber Cancer Institute, here we describe the development of the electronic New Patient Intake Questionnaire.

**Materials and Methods:**

The original New Patient Intake Questionnaire includes a review of symptoms, oncology history, family history, health behaviors, health and social status, health literacy and numeracy, which was modified for integration into the EHR using content determination, build and configuration, implementation, analytics, and interventions. The engagement of key stakeholders, including patients, clinical staff, and providers, throughout the development and deployment of the electronic Questionnaire was crucial to producing a successful tool. Continual modifications based on input of stakeholders (such as mode of tool deployment) were made to ensure the utility and usability of the tool for both patients and providers.

**Results:**

Implementation of the EHR-integrated electronic New Patient Intake Questionnaire improved collection of the PRD by increasing questionnaire accessibility for patients, while also providing all available data to clinicians and researchers. Careful consideration of the content and configuration of the questionnaire allowed for a successful, institute-wide implementation of the tool.

**Discussion:**

This effort demonstrates the feasibility of implementation of a system-wide electronic questionnaire, emphasizing the importance of iterative refinement to create a tool that is both patient-centric and usable for clinicians.

**Conclusions:**

The electronic New Patient Intake Questionnaire allows for systematic collection of the PRD, which should benefit cancer care outcomes through innovative care delivery and healthcare interventions.

## OBJECTIVE

The Patient Reported Data Program at the Dana-Farber Cancer Institute (DFCI) is tasked with implementing patient-oriented electronic data collection tools. The first of these was the New Patient Intake Questionnaire, assigned to all oncology patients at their first clinic visit. Herein, we describe development and implementation of the questionnaire.

## BACKGROUND AND SIGNIFICANCE

Many oncology centers employ intake questionnaires to gather demographic, lifestyle, and health information from new patients, which aids clinicians in patient assessment and development of a treatment plan. Health data reported directly by the patient on questionnaires are described as Patient Reported Data (PRD) and include subjective measures of functional and health status, quality of life, and symptom experience.^1^ Currently, PRD from questionnaires are integrated into the electronic health record (EHR) to screen for specific health conditions (such as genetic disorders) and monitor treatment-related symptoms. This integration allows for immediate retrieval of data and can augment collaborations between multidisciplinary care teams and patient-provider communication.^2–4^

Collecting and storing PRD in the EHR engages patients in their healthcare and further serves to meet standards set by the Health Information Technology for Economic and Clinical Health (HITECH) Act, enacted in 2009, which promotes meaningful implementation of information technology in healthcare.^5^ Collection and storage of electronic rather than paper-based PRD provides a standard repository for patient-level data that can be leveraged for population health initiatives with high patient acceptance,^3^^,^^6^ fewer data entry errors, easier questionnaire skip patterns, reduced effort from clinical staff to administer management of completed questionnaires, and more accurate and complete data.^6^

Here, we describe development of an EHR-integrated intake questionnaire for systematic collection of these PRD. We present factors that shaped the development, adaptation, and implementation of the tool from the perspective of key stakeholders. This electronic New Patient Intake Questionnaire (e-NPIQ) is utilized for clinical care, large-scale data collection, and screening patients for health and lifestyle factors that may impact their treatment. While intake questionnaires are common in clinical care, to our knowledge this is the only systematic, electronic, EHR-integrated intake questionnaire in use.

## MATERIALS AND METHODS

### Setting

DFCI is a National Cancer Institute-Designated Comprehensive Cancer Center based in Boston, Massachusetts with 7 established campuses located throughout New England. DFCI provides medical, surgical, and radiation oncology, as well as supportive oncology services for children and adults diagnosed with cancer or blood disorders.

### Development of the Electronic Intake Questionnaire

Prior to implementation of a new EHR in 2015, the intake questionnaire was a paper-based patient-survey. This 5-page questionnaire (deployed in 1989) was mailed to the patient with a request for completion prior to their initial visit.^7^ While the tool provided vital data to the primary physician, data were not available to extended members of the care team and required manual entry for aggregate analysis, thus limiting its utility.

Planning for the adoption of the Epic EHR in May 2014 presented an opportunity to improve the questionnaire and availability of data to all care team members at the DFCI. Therefore, a year-long stakeholder-engagement process was initiated to determine the feasibility of an EHR-integrated PRD tool that could be accessed through the EHR patient portal. The EHR integration effort would enable one seamless repository for clinical data to aid patient care and population-level research. Development of the tool consisted of 4 phases: (1) Content Determination, (2) Build and Configuration, (3) Implementation, and (4) Analytics and Interventions. Informed by the Consolidated Framework for Implementation Research,^8^ we collected qualitative feedback from key stakeholders on implementation outcomes including feasibility and acceptability as well as barriers and facilitators to implementation among at the patient, staff, clinician, and hospital administration levels.

### Content determination

A multidisciplinary panel of patient advocates and healthcare providers was engaged to reduce the question burden on patients and increase clinicians’ ease of interpretation. This 15-member panel was designated as the PRD workgroup and included 2 members of the Patient and Family Advisory Council as patient advocates, and 1 representative from the following operational/clinical teams: scheduling, ambulatory practice, radiology, nursing, pharmacy, infusion nursing, medical oncology, radiation oncology, surgical oncology, hematologic oncology, and psychosocial oncology. The team met at least once-a-week and reviewed the content of all questionnaires given to patients throughout DFCI, and the timepoints at which they were given to produce a spreadsheet of questionnaire data elements. Content and face validity of the questionnaire were also assessed by the PRD workgroup.

### Build and configuration

**Figure 1.**
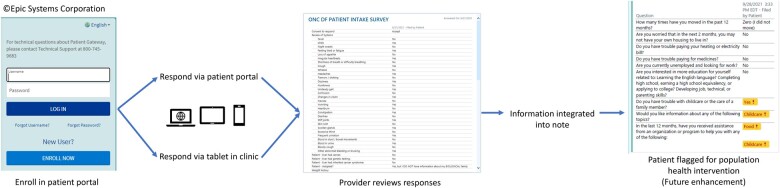
New Patient Intake Questionnaire data workflow.

The initial build of the e-NPIQ was conducted in partnership with the Mass General Brigham Integrated Healthcare Information System. Input from the Patient Portal Workgroup about questionnaire delivery workflow was reviewed by the DFCI Epic EHR Clinical Council (20 members in total) consisting of 1 member from each DFCI disease center, 4 Epic EHR application physician leaders, and the DFCI Epic EHR lead. Final approval was given by the DFCI Epic EHR Steering Committee (20 members including clinical administrators and clinical representatives from each department including nursing and pharmacy). The purpose of the workflow was to ensure consistency throughout the DFCI. Patient and clinical faculty expectations for the e-NPIQ were balanced with real-world capabilities of the EHR platform. Patient and clinician interfaces were limited to EHR constraints on font, color, and spacing. The user interface was limited to 1 color to indicate a threshold (yellow), 1 font, 1 size, and text formatting that did not allow for graphs or figures. The workflow is shown in Figure 1.

### Implementation

The e-NPIQ was incorporated into the EHR following consensus between hospital leadership and patient representatives on content and mode of delivery. A patient needs assessment, conducted in 2015, identified awareness, computer access, and the complexity of patient portal enrollment as the main barriers to completing the questionnaire.^9^ On May 30, 2015, the EHR was activated and the e-NPIQ was deployed via the patient portal. Three pilot studies optimized accessibility of the e-NPIQ and patient enrollment in the online patient portal: (1) a soft launch to train clinicians; (2) an intervention to increase enrollment; and (3) addition of a tablet for PRD.

### Analytics and interventions

Analytics involved extracting primary data from the EHR to allow for large-scale analysis that could support operational and research initiatives and secondary data for patient interventions. This required locating the data tables within the EHR model, developing data queries, and validating the data. Through an audit and feedback process, data were queried from the enterprise data warehouse (EDW), location and specification of data fields were identified, and requirements were developed to construct the data model. These included responses to the e-NPIQ and critical appointment data, such as emergency department visits, telephone interactions, and patient-level demographic information. Following validation, these data were made available as a subject-area mart in the EDW.

## RESULTS

### Content of the e-NPIQ

To be relevant across cancer types, the content of the e-NPIQ drew on the institute’s original paper intake questionnaire and from existing disease-center-specific questionnaires reportedly worded for a fifth-grade level of literacy, as recommended by the Patient Family Advisory Council. Questions were divided into 6 categories that were actionable for intervention, including symptoms, oncology and health history, and health literacy. A seventh category surveyed patient satisfaction with the questionnaire. Some response options are binary but many are multiselection appropriate to the domain (ie, PROMIS 10 or perceived health status). There are no areas for nonstructured data because the data are used as only a baseline capture of patient data with the full report of patient status is available in the clinic visit summary.

To reduce patient burden, the e-NPIQ was streamlined to remove redundant items, such as demographic data accessible elsewhere in the EHR, or items deemed not to add clinical value for an immediate intervention. The content was then revised to meet requirements of the Mass General Brigham Healthcare System and capabilities of the EHR system. For example, we excluded linking family history for genetic counseling and analysis due to limitations of the EHR in May 2015. All clinical directors and clinical department chiefs approved the final content (Table 1). This vetting process ensured patients were being asked the right questions at the right time and in the correct context for clinical care.

**Table 1.** Content of the final version of the electronic-New Patient Intake Questionnaire

Introduction			
Thank you for taking the time to complete the Dana-Farber New Patient Intake Survey. The information will help us provide care tailored to your needs during your appointment. Your responses will remain confidential and will only be shared with your care team to inform decisions about care. DFCI may review anonymous responses to help improve care for other patients. If you are unable to complete the survey prior to your visit, our staff will provide assistance at check-in. Click ACCEPT to get started.Note: Please do not use the survey to report health needs, such as pain, that require an immediate response. For any urgent needs, please call your doctor’s office. Please ask the New Patient Coordinators or your physicians and nurses if you have other questions.			
Category			
1. Review of symptoms			
Question			
Have you had the following symptoms in the past month?			
	Symptom	Answer	
	Fever	Yes	No
	Chills	Yes	No
	Night sweats	Yes	No
	Fatigue	Yes	No
	Loss of appetite	Yes	No
	Irregular heartbeats	Yes	No
	Shortness of breath or difficulty breathing	Yes	No
	Cough	Yes	No
	Wheeze	Yes	No
	Headaches	Yes	No
	Tremors/shaking	Yes	No
	Dizziness	Yes	No
	Numbness	Yes	No
	Unsteady gait	Yes	No
	Confusion	Yes	No
	Changes in vision	Yes	No
	Nausea	Yes	No
	Vomiting	Yes	No
	Heartburn	Yes	No
	Constipation	Yes	No
	Diarrhea	Yes	No
	Stiff joints	Yes	No
	Skin rash	Yes	No
	Swollen glands	Yes	No
	Excessive thirst	Yes	No
	Frequent urination	Yes	No
	Blood in stool/bowel movements	Yes	No
	Blood in urine	Yes	No
	Bloody cough	Yes	No
	Other abnormal bleeding or bruising	Yes	No
2. Oncology history			
Question	Answer		
Do you, or have you ever had cancer?	I don’t know	Yes	No
How many different types of cancer have you had?	1 type	2 types	3 types
What type of cancer did you have first?	Leukemia/Lymphomas/Blood disorders		
	Breast Disease/Cancer	Gynecologic	
	Gastrointestinal and Digestive		
	Head & Neck	Neurological (brain)	
	Sarcoma	Skin	
	Thoracic (chest/lung)		
	Genitourinary (kidney/prostate/testes)		
	Other Cancer Type		
*[Branched]* What type of leukemia/lymphomas/blood disorders?	Acute Leukemia	Chronic Leukemia	
	Hodgkin’s lymphoma	Multiple myeloma	
	Non-Hodgkin’s lymphoma		
	Other leukemia/lymphoma/blood disorder		
	I don’t know		
*[Branched]* What type of gastrointestinal and digestive cancer?	Gallbladder/bile ducts	Rectum	Stomach
	Esophagus	Colon	Pancreas
	GIST (stromal tumor)	Liver	Appendix
	Carcinoid/neuroendocrine		Anus
	I don’t know		
*[Branched]* What type of neurological (brain) cancer?	Meningioma	Glioblastoma	
	Astrocytoma		
	Other type of brain cancer		
	I don’t know		
*[Branched]* What type of sarcoma?	Leiomyosarcoma	GIST/Stromal	
	Other soft tissue sarcoma		
	Ewing’s sarcoma	Other bone sarcoma	
	Other sarcoma	I don’t know	
*[Branched]* What type of skin cancer?	Melanoma	Basal cell carcinoma	
	Squamous cell carcinoma		
	Premalignant skin cancer		
	Other skin cancer	I don’t know	
*[Branched]* What type of thoracic (chest/lung) cancer?	Lung: Nonsmall cell carcinoma		
	Lung: Small cell carcinoma		
	Neuroendocrine/carcinoid		
	Mesothelioma		
	Other lung/chest cavity cancer		
	I don’t know		
*[Branched]* What type of genitourinary (kidney/prostate/testes) cancer?	Prostate	Kidney (Renal)	
	Bladder/Ureter	Testicular	
	Penile		
	Other urinary (genitourinary) cancer		
	I don’t know		
*[Branched]* What type of gynecological cancer?	Ovary/Fallopian tube	Uterus	Cervix
How was your current cancer first detected?	I had a cancer screening test (eg, mammogram, colonoscopy) which led to evaluation and detection of the cancer.		
	I had a physical examination, including scans, blood tests, or other studies which led to evaluation and detection of the cancer.		
	I had a concern (or symptom) which led to evaluation and detection of the cancer.		
	I don’t know		
How old were you when you were first diagnosed with cancer?	9 or younger	10–19	20–29
	30–39	40–49	50–59
	60–69	70–79	80–89
	90 or older	I don’t know	
How old were you when you were diagnosed with your second cancer?	9 or younger	10–19	20–29
	30–39	40–49	50–59
	60–69	70–79	80–89
	90 or older	I don’t know	
How old were you when you were diagnosed with your third cancer?	9 or younger	10–19	20–29
	30–39	40–49	50–59
	60–69	70–79	80–89
	90 or older	I don’t know	
Have you ever had cancer genetic testing (blood test for inherited cancer syndrome)?		Yes	No
Have you ever been diagnosed with an inherited cancer syndrome?	I don’t know	Yes	No
*[If yes]* Which inherited cancer syndrome(s) was it?	BRCA (hereditary breast and ovarian cancer)		
	Lynch Syndrome (HNPCC or Colon Cancer Syndrome)		
	Polyposis: Familial Adenomatous Polyposis (FAP) or		
	Attenuated Familial Adenomatous Polyposis (AFAP)		
	Endocrine Syndrome (MEN1 or MEN2)		
	Li-Fraumeni Syndrome		
	Neurofibromatosis (NF1)		
	Paraganglioma and Pheochromocytoma Syndrome		
	Peutz-Jeghers Syndrome	Cowden Syndrome	
3. Family history			
Question	Answer		
Are you adopted?	Yes, and I have information about my BIOLOGICAL family		
	Yes, but I DO NOT have information about my BIOLOGICAL family		
	No		
*[If no or yes, has information]* Now think about your	Yes: 1 cancer	Yes: 2 cancers	
MOTHER: Has your MOTHER ever been diagnosed with cancer?			
*[Repeat cancer history question for first and second degree family]*			
4. Health behaviors			
Question	Answer		
In a typical week, how many times do you do vigorous/strenuous exercise? (heart beats rapidly, sweating) (eg, running, aerobics, cross country skiing, vigorous swimming, vigorous biking)	0	1	2
	3	4	5
	6	7	8
	9	10+	
In a typical week, when you do vigorous/strenuous exercise, what is the average duration per episode?	0–9 min	10–19 min	
	20–29 min	30–39 min	
	40–49 min	50–59 min	
	60+ min		
*[Repeat exercise frequency and duration question for moderate and light exercise]*			
How often do you feel really rested when you wake up in the morning?	Never	Rarely	
	Occasionally	Frequently	
	Almost Always		
How would you rate the quality of your diet over the past year?	Excellent	Very good	
	Good	Fair	Poor
How much do you want to change your diet?	Not at all	Not at all	
	Somewhat	Somewhat	
	Very much		
Do you regularly take vitamins, alternative medications, or herbal supplements?		Yes	No
*[If yes]* Which alternative medications and/or supplements, do you take regularly?	Herbal supplements	Multivitamins	
	Megavitamins	Macrobiotics	
	Nutritional supplement	Other	
*[If yes]* Not counting multivitamins, do you currently take Vitamin D (in calcium supplement or separately)?	Yes, most months	Yes, seasonal only	
	No		
*[If yes]* Enter the dose per day of Vitamin D that you	Less than 600 IU	600–900 IU	
Take.	1000–1500 IU	2000 IU or more	
	Don’t know		
Over the PAST WEEK, how OFTEN did you have pain?	Never	Rarely	
	Occasionally	Frequently	
	Almost Constantly		
Over the PAST WEEK, what was the SEVERITY of your pain at its worst?	None	Mild	
	Moderate	Severe	
	Very Severe		
Over the PAST WEEK, how much did pain INTERFERE with your usually or daily activities?	Not at all	Somewhat	
	A little bit	Very much	
	Quite a bit		
*[PROMIS-10 validated question*] In general, would you say your health is:	Excellent	Very good	
	Good	Fair	Poor
5. Health and social status			
Question	Answer		
How often have you been bothered by emotional problems such as feeling anxious, depressed, or irritable?	Never	Rarely	
	Sometimes	Often	Always
How would you rate your fatigue on average?	None	Mild	
	Moderate	Severe	
	Very Severe		
How would you rate your pain on average?	0	1	2
(0 = No pain, 10 = Worst imaginable pain)	3	4	5
	6	7	8
	9		
Do you currently live alone?		Yes	No
*[If no]* Who lives with you?	Spouse/Partner/Significant Other	Child(ren)	
	Parent(s)	Sibling(s)	
	Friend(s)/Roommate(s)	Nonfamily caregiver(s)	
	Other		
[*If children*] How many of the child(ren) living with you are Under the age of 18?	0	1	2
	4	5 or more	
[*If children*] Are you the primary caregiver for a family member with intensive care needs?		Yes	No
How difficult is it for you (your family) to meet monthly payments on your (family’s) bills?	No at all difficult	Not very difficult	
	Somewhat difficult	Very difficult	
	Extremely difficult		
6. Health literacy and numeracy			
Question	Answer		
How confident are you filling out medical forms?	Extremely confident		
	Somewhat confident	Quite a bit confident	
	Not at all confident	A little bit confident	
How confident are you in understanding medical statistics?	Extremely confident		
	Somewhat confident	Quite a bit confident	
	Not at all confident	A little bit confident	
Did you complete this survey on your own?		Yes	No
*[If no]* Who assisted you with completing this survey?	Family member or friend		
	A clinic staff member		A translator
	A healthcare professional		Other
Where did you complete this survey?	At home	In the clinic	
	Both		
7. Questionnaire Satisfaction			
Question	Answer		
*[If in the clinic or both]* On a scale of 1–10 how did you find your experience using this iPad to answer your questionnaire?	1	2	3
	4	5	6
	7	8	9
	10		
*[If in the clinic or both]* Would you use an iPad to complete a questionnaire in the future?	Yes, easy to use		
	Yes, with modifications		
	No, difficult to use		

The e-NPIQ content was carefully reviewed so no high-risk questions (ie, self-harm) were asked. The timing of the questionnaire was planned so concerning answers could be reviewed with the primary at the initial clinic visit and escalated with specialist referrals as appropriate. In this way, the e-NPIQ was tied into established mechanisms to ensure safety.

### Build and configuration

The build and configuration of the e-NPIQ allowed patients access through the online patient portal in the EHR. The patient can provide their responses to the questionnaire on any internet-enabled personal computer, tablet, or phone. Responses are stored in the EHR where clinicians can view the data, which can be extracted from the EHR and analyzed to inform population health interventions.

### Clinician data review

The e-NPIQ collected data from patients 24–72 h before their first clinic visit. The questionnaire was to be reviewed at the consultation, and the data documented within the consultation visit summary. The EHR-integrated format replaced a paper version, allowing for the first time its availability for the primary oncologist at the first clinic visit and for the rest of the care team in the EHR.

The system standard was to repeat the e-NPIQ at 365 days or with a new cancer diagnosis, but elements of the questionnaire could be updated by clinical care team at any time. Any e-NPIQ responses not accurately reported, or with a differing opinion from the clinician, were amended in the first clinic visit summary. However, patient responses were kept and acknowledged as truth separate from the clinician report. Patient report of health status was never used alone for billing or for assigning treatment, but instead used to inform a wholistic representation of patient health status and to trigger supportive care referrals when appropriate.

### Implementation

#### Pilot 1: Soft launch

It was determined a soft launch of the e-NPIQ would be a transitional period for clinicians and staff in this new aspect of standard care and feedback from the stakeholders suggested no undue burden should be placed on the coordinators for new patients. Therefore, prior to the soft-launch, group training was conducted with clinicians and coordinators. The pilot was conducted over 5 months, which allowed us to gain insight on patient and staff engagement with the e-NPIQ, usability of the platform through qualitative interviews with patients, and focus groups conducted with clinicians and staff. Postimplementation surveys with patients, staff, and clinicians were conducted at 30 days following the soft launch, which provided quantitative feedback about barriers to sustainability of the e-NPIQ. Patients reported difficulty accessing the questionnaire through the patient portal; clinical staff reported issues with usability due to the user interface. The soft launch resulted in the following workflow for delivering the e-NPIQ:

1. Coordinators encourage enrollment in the patient portal when scheduling the consultation visit for a new patient.
2. The e-NPIQ is then delivered via the patient portal with a request for completion at least 24 h prior to the new patient consultation.
3. Patient responses are transmitted to the EHR and fields in the first 6 categories are archived as discrete data, which can be accessed by all clinicians. Fields in the seventh category (questionnaire satisfaction) are archived as nondiscrete data.

#### Pilot 2: Patient portal enrollment intervention

**Figure 2.**
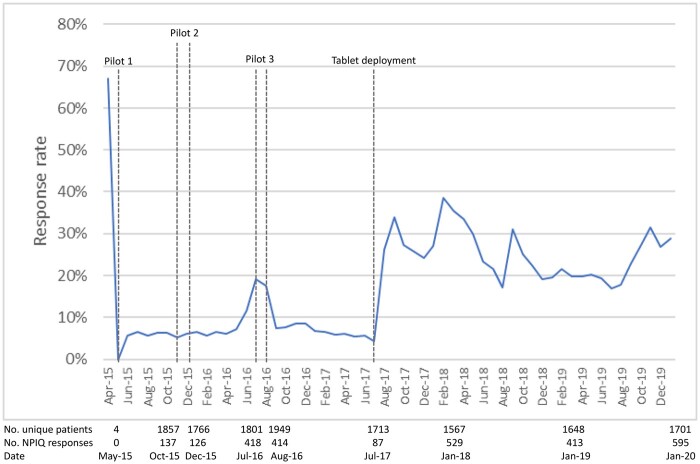
New Patient Intake Questionnaire response rate.

The PRD workgroup considered the soft launch successful because patient access to the e-NPIQ through the patient portal was consistently reliable, and the build and configuration of the questionnaire allowed for data storage within the EHR available for clinical assessment and research applications. However, the ultimate success of the e-NPIQ depended on patient response rates, defined as answering at least 1 question, and rates were only around 7% (Figure 2).

The patient portal is useful for engaging patients in their care. However, security concerns led the Mass General Brigham Healthcare System to require an activation code for creation of an account, which was a barrier to enrollment and lingered around 30% since the adoption of the EHR. In addition, the e-NPIQ and patient portal were only available in English and language limitations were and a challenge for questionnaire response rates. Several other barriers were reported by the 1019 oncology patients enrolled in the needs assessment survey: a lack of internet access (5%) or an internet-enabled device (13%), privacy concerns related to sharing health information electronically (10%), and, importantly, a limited awareness of the patient portal or its features (28%).

Because the patient portal was the only option for patient response, increasing patient portal enrollment was identified as the best means of increasing patient engagement. We initiation 3 interventions: (1) staff education, (2) staff-assisted patient enrollment support, and (3) independent patient enrollment support with a patient portal enrollment guide. These interventions increased portal enrollment rates from 46% to 52% across the Institute from November through December 2015.^9^

#### Pilot 3: Configuration of tablet devices

Although we had increased portal enrollment, this proved insufficient to meaningfully increase patient response rates. We consulted with the Patient and Family Advisory Council because the group had expressed concern that enrolling in the patient portal prior to the initial visit may be daunting when a patient is faced with a new cancer diagnosis or a request for another clinical opinion. Thus, a third pilot program was initiated which provided patients with a personal tablet configured with a software application to make it easier for patients to access the portal and respond to the e-NPIQ. Feedback from the 2015 patient needs assessment indicated a lack of computer access was a greater barrier than the complexity of patient portal enrollment.^9^

The Mass General Brigham Integrated Healthcare Information System staff devoted a significant effort to software configuration for the tablets, which run on Apple iOS software. However, the software application was built for Windows OS. Therefore, a Citrix Virtual Desktop Instance (VDI) interface was required.

Between July and August of 2016, a tablet device was provided to each new patient upon arrival at the gastrointestinal and thoracic oncology clinics of the DFCI. Tablet use increased response rates from approximately 18–51% for the 216 patients enrolled over the course of the pilot program. This significant increase resulted in the adoption of tablets as part of standard care for clinical practice across all departments at DFCI in August 2017. The response rate increased to 30% and continues to be sustained.

### Analytics and interventions

Data validation involved a cross functional team using operations and research resources, necessitated extensive mining of the EDW to accurately identify sources of data, and required substantial effort to ensure data validation over several months. To augment the value of PRD beyond strict clinical use, we focused on extracting this data for secondary analysis and targeted cohorts of patients for appropriate interventions. For example, we are developing an intervention to map social determinants of health to resource interventions and financial counseling. We received institutional approval to compile a data dictionary, which allows research teams (with IRB approval) to analyze secondary data and map social determinants of health.

### Ongoing staff engagement interventions

Several smaller steps have increased staff engagement, which initially was low based on personal reports from staff. Patient check-in, tablet distribution, and schedule follow up appointments conducted by Clinic Coordinators had a pivotal impact on the success of the tablet distribution in the clinic by reporting problems with check-in, technical glitches, and the importance that staff understand the significance of PRD collection. This input brought attention to the need for staff and faculty training sessions, which were undertaken to ensure that clinical personnel knew how to assign tablets at check-in and how to access the data in the EHR. Tablet assignment was streamlined to make distribution in the clinic easier, thus increasing the number of patients with access to the e-NPIQ via tablet. Initially, tablets were assigned by manual entry of a serial number. We updated this procedure by adding a drop-down menu, which has been further improved by scanning a QR code.

Clinic Coordinators were trained to engage with patients and facilitate clinic visits, including e-NPIQ data collection by tablet. Tablets were prepared for each patient prior to handoff to patients. The patient would then complete a 3-step digital privacy verification on the tablet, endorsed by the privacy office, before beginning the e-NPIQ.

Comments from 44 providers from the departments of gastrointestinal cancer, palliative care, and physician leaders from a process improvement workgroup noted the need for improvements in the usability of the provider views. They asked for a better display of questions, although they also highlighted beneficial ease of interpretation and documentation in the original provider view. Questions in the provider view were shortened and the flow was rearranged to be more logically ordered to increase visibility and readability. These changes underwent both functional and usability testing. Data collected in the e-NPIQ were moved to the patient summary screen, known as the Synopsis tab in the Epic EHR, for ease of access for all care team members, including cancer treatment staff such as infusion nurses, social workers, and nutritionists. The functionality of the e-NPIQ was demonstrated to staff and clinicians with departmental “roadshows” in the Fall of 2020, which recapped the launch of the e-NPIQ, reviewed the data being collected, and solicited discussions on strategies tailored for the individual data needs of each department.

### Patient response to the e-NPIQ data collection tool for PRD

Our first evaluation of data following deployment of the e-NPIQ via tablet distribution in clinics included response rates (as described above) and the readiness of patients to complete electronic surveys. To quantify patient readiness, 2 questions on usability and engagement were included in the e-NPIQ (Table 1, category 7). When asked to rate the experience of using the tablet on a scale of 1–10, 82% of patients rated the experience as an 8 or higher, with 52% of patients overall reporting an experience of 10. When asked if they would use a tablet in the future, 97% responded yes, including 92% of patients aged ≥70 years (*n* = 86). Patients reported that they were able to complete all questions on the e-NPIQ in less than 10 min.

## DISCUSSION

EHR-embedded questionnaires allow for the systematic collection of PRD by increasing enrollment and access to the questionnaire for patients, and data access for clinicians for population health interventions. This EHR-integration creates a more complete and accurate medical history that approaches the patient-centered intent of the HITECH Act.^10^ However, there is currently a gap on how to successfully deploy systematic PRD data collection tools in standard care.

The development of the e-NPIQ for PRD at the DFCI demonstrates the feasibility of implementing electronic collection of PRD in standard clinical practice and emphasized the importance of continual iterative refinement of content and means of delivery of the e-NPIQ, and display of data. This flexibility is required to meet the changing needs of patients, clinic staff, and clinicians within the larger context of the healthcare system’s data policies and capabilities of the EHR. Content that is relevant and actionable has been continually cited as an important keystone when building effective PRD questionnaires.^11–13^ The continual refinement of the intake questionnaire’s configuration after implementation was critical for building PRD tools that could be incorporated into clinical practice, allowing us to create an intake questionnaire with usability that met the demands of clinical staff and healthcare providers.^12^^,^^13^ The final steps developing the e-NPIQ included understanding how to extract the data from the EHR. Future innovations will apply that data to inform the development and implementation of routine care population health interventions in clinical settings. The data from the NPIQ will be available to researchers (with IRB approval) for analysis.

Challenges to implementing the tool at the patient level included insufficient access to the patient portal, which limited response rates and at the staff and provider level included workflow barriers that impeded tablet assignment, and cluttered data display that reduced the usability of the data for standard care. These obstacles echo barriers to develop an electronic tool to collect PRD reported by other groups: how to use technology effectively to disseminate the questionnaire to patients, securely transfer data to the EHR, format the questionnaire and display the data, and obtain buy-in from the institution.^2^^,^^12^^,^^14^

A formal assessment of barriers to patient participation required drawing on technological logistics of patient access to the portal, enrollment, and a tablet intervention. We reduced barriers by ongoing training for clinical staff and a streamlined workflow for tablet assignment. Data display was also continuously updated to meet clinician needs for readability and usability. The iterative development of not just the tool’s functionality but more fundamentally its content has led to ongoing endorsement of the patient-reported data to inform oncology care beyond patient-reported outcomes, adding risk stratification, social determinants of health, and pertinent patient history.

There is room for improvement of the e-NPIQ. Future work will examine other strategies to improve the utility of the tool for patients and clinicians. We are currently exploring the addition of text messaging as a third modality for sending questions and receiving patient responses. We also hope to increase patient awareness of the e-check-in process for clinic visits available through the patient portal, where patients are prompted to complete questionnaires. In the future we hope to completely circumnavigate the patient portal by allowing patients to answer questionnaires on their personal mobile devices without accessing the portal.

Expanding language beyond English for Spanish-speaking patients is a high priority and additional plans are in place to included Russian, Portuguese, and Arabic. Aggregate data results will periodically be shared with patients, clinicians, and the research community to demonstrate the value of the e-NPIQ and other tools for collecting PRD. Privacy and data use concerns will be addressed by reviewing and reinforcing the scope of data collected and who is granted permissions to access data files. We are developing several tertiary population health interventions to integrate into routine care based on patient responses, including resource matching, referral to genetic counselors based on family cancer history, and clinical trial education, which will increase the impact of the e-NPIQ.

### Limitations to development of the e-NPIQ

There were several limitations during development and implementation of the e-NPIQ. The most prominent challenge was constraints of the EHR framework on the user interface for patients and clinicians. However, feedback from patient and clinical stakeholders helped iteratively refine both interfaces. Next, patient access was increased by targeting patient portal enrollment. We then deployed the tablet, which allowed patients to access to the e-NPIQ and bypass portal enrollment, leading to a 27% patient response rate across the entire institute. Future iterations of the questionnaire will be available in other languages to increase access. The provider view was improved by training clinicians in retrieval of questionnaire responses, refinement of the data display within the EHR, and truncating question stems. Organizing responses by categories improved interpretation for clinicians and adding the data to the patient summary screen of the EHR increased access for all care team members.

## CONCLUSIONS

Validation of the e-NPIQ data established health metrics and expanded the utility of an electronic PRD for population health data analysis of targeted interventions. Electronic documentation of patient-reported data can enhance patient derived clinical, genomic, and pathologic data. Our experience building the e-NPIQ as part of an EHR is intended to allow other institutions choosing to design an e-PRD to build on lessons learned during development a PRD tool, which can to further cancer care delivery and improve care outcomes.

## FUNDING

We acknowledge the support and funding of the Dana-Farber Cancer Institute Patient Reported Data Program (all coauthors).

## AUTHOR CONTRIBUTIONS

NJM developed the project concept and directed the work. JLFC and MM contributed to data acquisition and analysis. EKH and JLFC drafted the manuscript. All authors critically revised the work for important intellectual content, gave final approval of the version to be published, and agree to be accountable for all aspects of the work including ensuring accuracy and integrity.

## Data Availability

The data underlying this article cannot be shared publicly due to HIPAA. The data will be shared on reasonable request to the corresponding author.
